# A Multi-Step Grasping Framework for Zero-Shot Object Detection in Everyday Environments Based on Lightweight Foundational General Models

**DOI:** 10.3390/s25237125

**Published:** 2025-11-21

**Authors:** Ruibo Li, Tie Zhang, Yanbiao Zou

**Affiliations:** School of Mechanical and Automotive Engineering, South China University of Technology, Guangzhou 510641, China; lrb18575737093@163.com (R.L.); ybzou@scut.edu.cn (Y.Z.)

**Keywords:** robotic grasping, object detection, image segmentation, zero-shot object, model quantization

## Abstract

Achieving object grasping in everyday environments by leveraging the powerful generalization capabilities of foundational general models while enhancing their deployment efficiency within robotic control systems represents a key challenge for service robots. To address the application environments and hardware resource constraints of household robots, a Three-step Pipeline Grasping Framework (TPGF) is proposed for zero-shot object grasping. The framework operates on the principle of “object perception–object point cloud extraction–grasping pose determination” and requires no training or fine-tuning. We integrate advanced foundational models into the Object Perception Module (OPM) to maximize zero-shot generalization and develop a novel Point Cloud Extraction Method (PCEM) based on Depth Information Suppression (DIS) to enable targeted grasping from complex scenes. Furthermore, to significantly reduce hardware overhead and accelerate deployment, a Saturated Truncation strategy based on relative information entropy is introduced for high-precision quantization, resulting in the highly efficient model, EntQ-EdgeSAM. Experimental results on public datasets demonstrate the superior inspection generalization of the combined foundational models compared to task-specific baselines. The proposed Saturated Truncation strategy achieves 3–21% higher quantization accuracy than symmetric uniform quantization, leading to 3.5% model file compression and 95% faster inference speed for EntQ-EdgeSAM. Grasping experiments confirm that the TPGF achieves robust recognition accuracy and high grasping success rates in zero-shot object grasping tasks within replicated everyday environments, proving its practical value and efficiency for real-world robotic deployment.

## 1. Introduction

With the advancement of household service robotics, robots are becoming increasingly capable of enhancing users’ quality of life through the ability to grasp common household objects. However, in daily life, a more robust and lightweight robotic grasping framework is required because of changing environments, lighting variations, and limited hardware resources. Additionally, the ability to accurately distinguish between different types of household items to respond to user needs is one of the criteria for evaluating the quality of robotic services. Therefore, compared to industrial grasping scenarios, household service robots impose higher demands on the generalization capabilities of the deployed grasping framework. The primary research focus of this paper is: Based on the principle of “object perception–object point cloud extraction–grasping pose determination” and the strong generalization capabilities of foundational general models, we propose a robot grasping framework that is object-generalizable, lightweight, and robust. This framework addresses the hardware resource demands of control systems and enables zero-shot grasping of objects in everyday environments.

The robot requires embedding a grasping framework within its control system to achieve object localization and control the robot to execute grasping actions. Research on robot grasping frameworks primarily falls into two categories: single-step grasping frameworks based on end-to-end networks and multi-step grasping frameworks based on segmentation. The former primarily constructs grasping generation networks based on certain grasping metrics, directly generating grasping poses through the network. For example, Fang et al. [1] proposed an end-to-end network for grasp pose prediction from point cloud inputs, which decouples the learning of grasp approach direction and configuration parameters. Based on Fang et al.’s work, Wang et al. [2] proposed GSNet to generate robust 6D grasp poses for scene point clouds. GSNet is a two-stage end-to-end network incorporating per-point, scene-level grasp scores and a grasp sampling strategy. Based on GSNet, AnyGrasp [3] defined grasp stability scores in terms of the distance between the object’s center of mass and the gripper plane. Trained on real-world data of everyday objects, AnyGrasp achieved human-comparable performance in zero-shot object grasping experiments.

Single-step grasping frameworks employ end-to-end networks trained on grasp metrics, enabling them to directly predict robust grasp poses. However, when grasping accuracy is not achieved, identifying the root cause of errors becomes challenging due to the single-step nature of the process. Furthermore, these methods rely on high-quality datasets with dense point cloud annotations, which incur substantial human labor and time costs. In contrast, multi-step grasping frameworks utilize RGB-D images as input, integrating instance segmentation with diverse grasp pose determination methods. These frameworks divide the grasping problem into three subtasks: object recognition and localization, pose estimation, and grasp determination. Various methods commonly employed to tackle the three sub-tasks have been proposed in studies. Ainetter et al. [4] proposed a CNN-based deep neural network. The network generates high-quality grasp detection results for specified objects by utilizing precomputed grasp detection and semantic segmentation information. Duan et al. [5] proposed the Multi-task Semantic Grasp Convolutional Neural Network (MSG-ConvNet) to enable robust grasping in heavily occluded scenes. Liu et al. [6] developed a comprehensive semantic grasping framework that combines few-shot semantic segmentation with grasp pose detection, facilitating grasping for specific object categories while demonstrating a certain level of generalization for zero-shot objects within those categories. These methods utilize RGB-D images as inputs, reducing reliance on high-quality dataset annotations for model training. However, their complex network structures demand significant computational resources. Additionally, while these methods focus on enhancing grasping accuracy, they do not address the generalization of grasp detection to zero-shot objects. Moreover, the aforementioned grasping frameworks may experience malfunctions when operating under extreme everyday environments (e.g., haze, dark lighting). To address these potential failures, Wang et al. [7] introduce DNMGDT, a network that jointly leverages synthetic and real hazy images within a shared-parameter architecture. The method incorporates multiple prior-based pseudo-clean images and employs an adaptive weighting strategy to emphasize reliable regions while suppressing artifacts. In addition, a physical-model-guided domain transfer mechanism is used to reduce the domain gap between synthetic and real scenes. This combination significantly improves generalization and dehazing performance on real-world hazy images. And they also introduce a weakly supervised dehazing method based on physics-based decomposition (PBD) [8], which estimates illumination and haze-related parameters to produce a physically consistent dehazed image. A discrete wavelet discriminator further enhances generalization to real scenes using unpaired clear images. This approach avoids handcrafted priors and effectively reduces residual haze and distortions, achieving strong performance across real-world datasets.

With advances in deep learning, foundational general models have increasingly demonstrated powerful learning capabilities. These models utilize Transformer, a versatile and scalable foundational network module, as the main component of their backbone network. By fully learning from large-scale datasets, foundational general models achieve powerful zero-shot inference performance. Recently, several Vision–Language–Action (VLA) models have demonstrated the ability to achieve spatial object perception and exhibit strong generalization capabilities, such as Google’s RT-1 [9] and RT-2 [10], as well as the pi series models from Physical Intelligence [11,12]. Trained on Internet-scale data and leveraging Transformer-based architectures, these VLA models can fully learn extensive knowledge about “users providing visual information and textual requests, with robots executing corresponding actions.”. This enables robots to address diverse task requirements across various scenarios. However, these VLA models exhibit low modularity and poor interpretability, making it difficult to optimize or replace individual components. Furthermore, their parameter counts often reach billions (e.g., RT-2 has 55 billion parameters), necessitating deployment in cloud platforms to maintain acceptable computational time costs. When deployed within robotic control systems, large VLA models may struggle to guarantee real-time performance. In addition to VLA models, Wang et al. [13] propose a Capsule Attention Network (CAN) that integrates capsule activity vectors with attention mechanisms for hyperspectral image classification. An attention-based feature extractor and a self-weighted capsule mechanism jointly enhance spectral-spatial representation. Experiments on four datasets demonstrate improved accuracy over prior methods with lower computational cost. And they also introduce a large-scale HSI clustering framework based on doubly stochastic graph learning [14], which jointly learns a projected spectral space and both pixel-anchor and anchor-anchor graphs. The doubly stochastic constraint ensures more reliable affinity estimation and cluster indicators. Their method also achieves linear complexity, making it well suited for large hyperspectral datasets, and shows clear performance gains over prior clustering approaches. Moreover, Wang et al. [15] propose a multi-order graph clustering framework based on dynamic low-rank tensor approximation. Their method enriches graph inputs using high-order proximities and dynamically extracts consistent structural information across multiple graphs. A doubly stochastic graph fusion strategy is then used to produce a clean and symmetric affinity graph whose connectivity directly yields cluster assignments. This approach achieves state-of-the-art clustering performance and generates highly structured graphs on benchmark datasets.

To enhance the generalization of multi-step grasping frameworks, researchers have employed Transformer-based foundational models to improve zero-shot object recognition and perception, such as Grounding DINO [16] and Segment Anything Model (SAM) [17]. Ceschini et al. [18] proposed a two-step, training-free grasping pipeline. In the first step, they utilized bounding boxes generated by Mask R-CNN [19] as prompts and employed SAM-H to refine segmentation masks. In the second step, they determined grasp points using 2D segmentation masks and depth images, achieving a balance between grasping accuracy and computational efficiency. Li et al. [20] employed Grounding DINO and SAM to segment object masks, integrating these models with their proposed Sim-Suction-PointNet network to generate 6D poses for target objects, effectively solving the challenge of grasping zero-shot objects in cluttered environments. Noh et al. [21] proposed GraspSAM, a framework built upon the zero-shot capabilities of the SAM by adding an adapter for the image encoder, a decoder with several additional MLP layers, and lightweight tokens learning to enable object mask and grasp map prediction. These methods, utilizing the zero-shot perception abilities of Grounding DINO and SAM, effectively tackle the generalization challenge for zero-shot objects. However, SAM’s numerous parameters, low computational efficiency, and high resource demands result in significant hardware consumption for both computation and storage. To address these challenges, lightweight strategies are needed to reduce SAM’s resource usage.

For SAM’s lightweight optimization, researchers initially explored using lightweight models and retraining them on the SA-1B dataset [17]. Zhao et al. [22] employed the CNN-based YOLOv8-Seg, reducing the parameters by 89.4% compared to SAM-H while maintaining 86.6% of the original model’s accuracy. Xiong et al. [23] replaced ViT-H with ViT-Tiny, resulting in a 2.57% accuracy drop while maintaining only 1.59% of the original model’s parameters. While these approaches largely fulfill the lightweight requirements for SAM, training on the full SA-1B dataset over multiple cycles still incurs substantial computational costs. Therefore, some studies have proposed replacing SAM’s heavy image encoder with a lightweight version and training it using Knowledge Distillation. Zhang et al. [24] proposed EfficientViT-SAM, which employs EfficientViT [25] to extract knowledge from ViT-H and achieves lightweighting by replacing hardware-unfriendly softmax operations with the Multi-Scale ReLU Linear Attention. EfficientViT-SAM-XL1 retains only 31.7% of SAM-H’s parameters while incurring a 20.62% accuracy loss. Zhang et al. [26] proposed an encoder-only distillation strategy, transferring knowledge from ViT-H to TinyViT to develop MobileSAM. With only 10.83M parameters, MobileSAM achieves a 98.4% parameter reduction compared to SAM-H, accompanied by a mere 1.49% accuracy loss. Unlike the approaches of Cai and Zhang, Wang et al. [27] employed a pure CNN-based RepViT as the image encoder and utilized the same distillation strategy as MobileSAM. This method reduced SAM-H’s parameters by 95.75% while maintaining 95.77% accuracy. Based on Wang et al.’s work, Zhou et al. [28] proposed a dynamic prompt-in-the-loop strategy and utilized a much pure CNN architecture for image encoding, leading to the development of EdgeSAM. According to [29], EdgeSAM contains only 9.58M parameters and incurs a minimal accuracy drop of 1.15%, establishing it as the current SOTA method among SAM variants. These studies indicate that Knowledge Distillation, compared to retraining, effectively reduces training time and computational resource demands while achieving a more lightweight network with less accuracy degradation. Some studies address more resource-constrained deployment conditions and have utilized post-training quantization to SAM for lightweight optimization without any training. Liu et al. [30] proposed a mixed-precision post-training quantization method for ViT, which utilizes similarity-aware quantization to determine simplified intervals and incorporates ranking loss for attention maps, achieving partial Int8 quantization of the ViT module. Lin et al. [31] addressed the unquantized LayerNorm layers and softmax operations in Liu et al.’s work by proposing Power-of-Two Factor (PTF) quantization and Log-Int-Softmax(LIS) quantization, achieving full Int8 quantization of the ViT module. Yuan et al. [32] proposed a twin uniform quantization method to minimize quantization error, utilizing Hessian-guided metrics to determine quantization parameters. This approach addressed the issue of non-uniform distributions in softmax and GELU activations, resulting in a smaller accuracy drop. In contrast to the above methods, which focus solely on quantizing the ViT module, Lv et al. [33] proposed PTQ4SAM, a post-training quantization framework for the entire SAM. By utilizing Bimodal Integration (BIG) and Adaptive Granularity Quantization (AGC), it addresses the bimodal distribution in post-Key-Linear activations and distribution differences in Attention mechanisms, achieving only a 0.5% accuracy drop and a 3.9× speedup. However, in practical deployment, although these methods achieve the conversion from floating-point to lower-bit precision and enable certain speedups on CPUs through quantization toolkits, they are not supported on GPUs. Therefore, further research is needed to develop GPU-compatible quantization methods to achieve lightweight deployment of SAM in robotic control system.

Inspired by the studies mentioned above, this study focuses on home service robot applications and proposes a lightweight TPGF. The framework is designed to grasp zero-shot objects in everyday environments with limited hardware resources. Specifically, the proposed TPGF comprises object perception, object point cloud extraction, and grasp pose determination. Specifically, the framework constructs the generalized OPM using foundational general models, and utilizes the Saturated Truncation strategy for lightweight optimization. It utilizes the DIS method to efficiently extract object point clouds and generates robust grasp poses through the Grasp Pose Determination Module (GPDM).

The main contributions of this study are as follows:

1. A novel and efficient Three-step Pipeline Grasping Framework (TPGF) is proposed. This framework addresses the critical challenges of convenient deployment, zero-shot object perception in everyday environments, and low-latency performance in service robotics. By modularly integrating foundational general models (Grounding DINO and EdgeSAM) and a pre-trained grasp network (AnyGrasp), the TPGF achieves robust grasping of zero-shot objects in cluttered scenes without requiring additional training or fine-tuning.
2. A Saturated Truncation strategy based on minimizing relative information entropy is proposed. This strategy specifically targets the limited computational and storage resources of household service robots. It significantly enhances the accuracy of Int8 quantization for the EdgeSAM, effectively reducing hardware overhead and enabling EntQ-EdgeSAM to achieve a 95% faster inference speed compared to the SAM used in existing grasping frameworks.
3. The practical value and robustness of the TPGF through comprehensive experimentation are validated. Experimental results demonstrate the superior generalization capability of our Object Perception Module (OPM) in zero-shot object perception tasks. Furthermore, the TPGF demonstrates high recognition accuracy and grasping success rates in replicated everyday environments, providing an efficient and reliable solution for practical service robot deployment.

The contents of the following sections are as follows. In Section 2, a TPGF for zero-shot objects in everyday environments is proposed. The limitations of traditional quantization methods in practical deployment are analyzed, and a Saturated Truncation strategy is proposed to optimize the data distribution before quantization and reduce quantization errors. In Section 3, the public dataset containing real-world scenarios is preprocessed first. Comparative experiments are conducted to validate the generalization performance of the OPM and the effectiveness of the Saturated Truncation strategy. In Section 4, a grasping platform is constructed based on a robot and vision system, and everyday environments with varying complexities are replicated to verify the generalization and robustness of the proposed grasping framework. In Section 5, we discuss the limitations and future works. In Section 6, the conclusions of this study are discussed.

## 2. Proposed Three-Step Pipeline Grasping Framework

**Figure 1:**
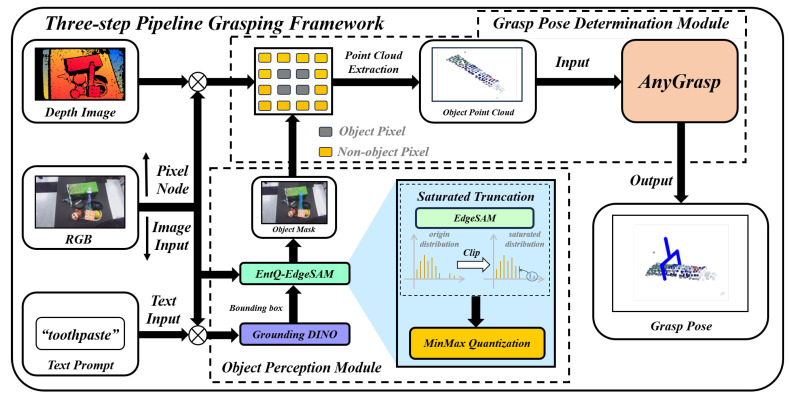
Overview of the Three-step Pipeline Grasping Framework.

Household service robots face several challenges when recognizing target objects and performing grasping tasks in complex everyday environments, including zero-shot objects, cluttered object placements, and limited computational resource in the robotic control system. To address these challenges, the proposed TPGF operates as shown in Figure 1.

The proposed TPGF offers distinct advantages in real-world deployment compared to monolithic end-to-end Vision–Language–Action (VLA) models, such as RT-2. Unlike VLA models which rely on massive, general-purpose transformer architectures, the TPGF adopts a modular design that explicitly separates perception, segmentation, and grasp determination. This design translates directly into superior real-time performance and reduced deployment complexity. Real-time performance is achieved by integrating specialized, highly optimized components, such as the EntQ-EdgeSAM model, which achieves an inference time of just 3.086 ms on standard GPUs. This highly efficient pipeline drastically contrasts with VLA models, which often have billions of parameters and suffer from high latency, frequently requiring non-edge GPUs or cloud connection. For deployment complexity, VLA models present a high barrier due to their fixed, immense file size and the difficulty of optimizing them for edge devices. In contrast, the TPGF, specifically through our Saturated Truncation strategy, enables deployment with a significantly reduced storage footprint and lower hardware overhead suitable for resource-limited household service robots. Furthermore, the modularity ensures (1) interpretable intermediate results (e.g., explicit object masks and 6D poses) and (2) straightforward, plug-and-play module replacement, capabilities generally absent in fixed, end-to-end VLA models.

The grasping framework utilizes real-time RGB images, depth images, and textual information about the target object as input. Grounding DINO generates a bounding box for the target object using the RGB image and textual information. The bounding box is then used as a prompt and fed into EdgeSAM along with the RGB image to generate an object mask. To reduce the computational resource requirements and storage costs of EdgeSAM, its image encoder is quantized using the Saturated Truncation strategy, resulting in EntQ-EdgeSAM. In the PCEM, the mask is used to filter depth information, ensuring that only the target object’s point cloud is generated after aligning the depth map with the RGB image. Finally, AnyGrasp generates a 6D grasp pose for the object for robot to execute the grasping task.

### 2.1. Object Perception Module Based on Foundational General Models

The proposed TPGF relies on the OPM with zero-shot generalization capability as the foundation for zero-shot object grasping. To enhance object perception accuracy in everyday environments, the OPM first utilizes a zero-shot object detection method to generate object region proposals and then employs a class-agnostic instance segmentation method to extract pixel-level object features for subsequent steps.

However, traditional object detection methods depend on pre-defined class supervision signals during training, limiting their detection results to specific classes. This constraint restricts their generalization capability to objects outside these predefined categories. To address this limitation, we utilize Grounding DINO, an open-set object detector, for zero-shot object detection. Unlike traditional closed-set detection methods, Grounding DINO utilizes an open-set detection scheme where the detected categories are provided externally, rather than predetermined. The object region proposals and their corresponding categories are directly generated by a network that integrates textual and visual information, rather than being derived through logits-based category label matching.

**Figure 2:**
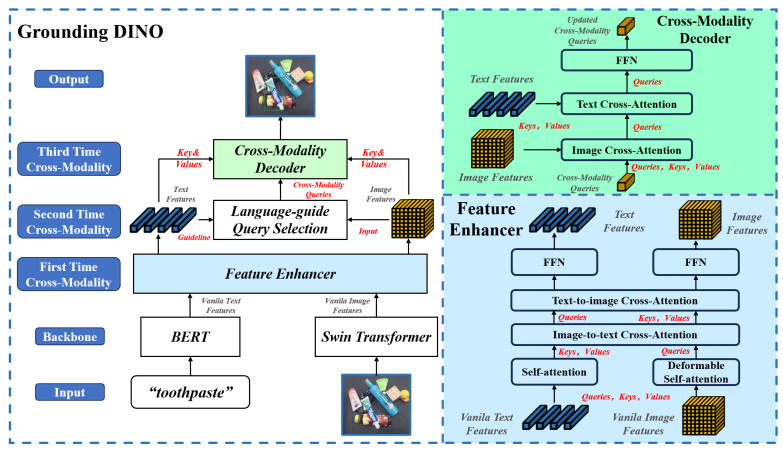
Grounding DINO Network Architecture [16]. The left side shows the overall network diagram, while the top and bottom on the right depict the structures of the cross-modal decoder and the feature enhancer, respectively.

Grounding DINO achieves zero-shot object detection capability through three rounds of cross-modal feature fusion between text and image. The process of handling the object’s textual information and image in Grounding DINO is depicted in Figure 2. A text backbone using the pre-trained language model BERT [34] extracts text features from the textual information, while an image backbone based on Swin Transformer [35] extracts image feature from the image. These features are then fed into a feature enhancer module. In this module, the text features and image features are separately processed using self-attention and Deformable self-attention. The two features then serve as queries and undergo cross-attention calculations with each other, completing in the first round of cross-modality feature fusion. Finally, both features utilize a Feed-Forward Network(FFN) to generate the fused feature vectors. Within the Language-Guided Query Selection module, the maximum similarity between each image feature vector and all text feature vectors is calculated according to Equation (1) [16], completing the second round of cross-modality feature fusion:

(1) $$Sim=\max_{1⩽i⩽N_{I}}\left(X_{I_{i}},x_{T}^{T}\right)$$

where Sim represents the similarity, $X_{I_{i}}\in R^{1\times d}$ represents one of the image features $X_{I}\in R^{N_{I}\times d}$, $X_{T}\in R^{N_{T}\times d}$ represents all text features, *d* represents the feature dimension, and T represents the transpose of the matrix. Then, based on the maximum similarity, the image feature index *i* is used to extract the corresponding image feature as the initial query, which is fed into multiple cross-modal decoders. In each cross-modality decoder, the text features and image features undergo the third round of cross-modality feature fusion. The initial query first performs self-attention calculations. Then, as a query token, it sequentially performs cross-attention calculations with both the image and text features to achieve modal fusion and modal alignment. Finally, it utilizes a Feed-Forward Network to output the object bounding box, representing the object region proposal information.

After obtaining the image region proposals of the target object, instance segmentation is performed to extract pixel-level object features. In this study, the foundational class-agnostic segmentation model EdgeSAM is employed as the instance segmentation method. In the OPM, EdgeSAM takes a real-time RGB image as input to the image encoder to generate image embeddings. The object region proposals generated by Grounding DINO serve as sparse prompts, which are fed into the prompt encoder to generate prompt embeddings. Finally, the mask decoder processes these embeddings to generate binary masks representing pixel-level object features.

This combination significantly improves generalization in zero-shot object perception. Grounding DINO provides high-level semantic grounding from textual cues, allowing detection of unseen categories. And EdgeSAM contributes fine-grained pixel-level precision with robust edge awareness. Their complementary strengths enable accurate and category-agnostic object segmentation without additional training, which forms a key foundation for generating reliable object point clouds in downstream grasp pose detection.

Compared with using either model alone, the combination ensures both semantic relevance and pixel-level precision, effectively bridging the gap between open-set object detection and instance-level segmentation.

### 2.2. Saturated Truncation Strategy Based on Minimizing Relative Entropy

Although EdgeSAM achieves some lightweight optimization under sufficient computational resources [28], its real-time performance falls short of grasping task requirements when processing high-resolution images (for example, 1920×1080 or higher) or operating under limited computational resources, ultimately degrading the robot’s service quality. Additionally, the limited storage capacity of household service robot restricts the robot to deploy lightweight model files. To address these challenges, the Saturated Truncation strategy is utilized to perform Int8 quantization on EdgeSAM.

For post-training quantization, the quantization step size is necessary to be determined based on the quantization bit width, and a quantizer is utilized to map floating-point values to the nearest quantization levels. A widely used quantization method supported by GPU acceleration is Symmetric Uniform Quantization [36]:

(2) $$Q\left(x|b\right)=\mathrm{Clip}\left(\mathrm{Round}\left(\frac{x}{s}\right),-2^{b-1},2^{b-1}-1\right)$$

where $Q\left(x|b\right)$ represents the quantizer, *x* denotes the variable to be quantized, *b* is the quantization bit width, Clip denotes the clipping operation on the values outside the quantization interval, and Round represents the rounding operation. The quantization step size *s* is determined by the upper bounds *u* and lower bounds *l*, which are usually the maximum and minimum values of the data:

(3) $$l=\min\left(x\right),u=\max\left(x\right)$$

(4) $$s=\frac{u-l}{2^{b}-1}$$

After quantized inference, the quantized values need to be dequantized to restore the model’s functionality. The dequantization formula for Symmetric Uniform Quantization is

(5) $$x=(Q\left(x|b\right)-\left(-2^{b-1}\right))*s$$

Due to the linear mapping nature and fixed quantization step size of Symmetric Uniform Quantization, when the variable follows a non-saturated distribution (where a few values lie near the interval boundaries and most values cluster around the midpoint), the data cannot be evenly distributed between across quantization levels, resulting in a significant difference between the pre- and post-quantization data distribution. This discrepancy prevents the dequantized data from fully recovering the original floating-point values. Therefore, a Saturated Truncation strategy is proposed to enhance the quantization accuracy of non-saturated distributed data in this study. The strategy dynamically calculates a truncation threshold *t* for the output variable at each computational step in the network. By using the truncation interval [−t,t], the data is evenly distributed across quantization levels, minimizing the discrepancy between pre- and post-quantization data distributions and ultimately reducing quantization error. Based on the above analysis, the mathematical model for the Saturated Truncation strategy is established as follows:

(6) $$\begin{array}{c} \begin{array}{cc} \; & \min_{t>0}\;\;\mathrm{Loss}\left(f\left(x_{q}\right),f\left(x\right)\right)\\ \; & s.t.\begin{array}{c} X_{q}=Q\left(f_{t}\left(x\right)|b\right)\\ f_{t}\left(x\right)=\mathrm{Clip}\left(x,-t,t\right) \end{array} \end{array} \end{array}$$

where $x_{q}$ represents the origin data *x* after quantization, f(x), f(q) respectively denote the distribution of data before and after quantization, Loss represents the data distribution loss function, and $f_{t}\left(x\right)=\mathrm{Clip}\left(x,-t,t\right)$ represents the clipping operation performed on *x* using the truncation interval [−t,t].

To precisely measure the discrepancy in data distribution before and after quantization, we utilize the relative Entropy (also known as Kullback–Leibler Divergence), denoted as $D(X||X_{q})$.

Relative Entropy is an effective metric for quantifying how much “information is lost” when the original high-precision data distribution (*X*) is approximated by the new low-precision distribution ($X_{q}$). Minimizing the relative Entropy means the truncation threshold *t* is selected to make the quantized distribution as statistically close as possible to the original one.

By using the relative Entropy as the specific objective function for minimization, the complete mathematical model for the Saturated Truncation strategy is obtained:

(7) $$\begin{array}{c} \begin{array}{cc} \; & \min_{t>0}\;\;D(X||X_{q})\\ \; & s.t.\begin{array}{c} X_{q}=Q\left(f_{t}\left(x\right)|b\right)\\ f_{t}\left(x\right)=\mathrm{Clip}\left(x,-t,t\right) \end{array} \end{array} \end{array}$$

Based on the Saturated Truncation strategy described above, the post-training quantization process for EdgeSAM is outlined as follows: First, FP32 inference is performed on EdgeSAM using a calibration set consisted of images. Subsequently, the distribution of the activation outputs’ absolute values at each layer of the image encoder is analyzed across several statistical intervals spanning the range between the minimum and maximum values. The midpoint of each statistical interval is selected as the corresponding truncation threshold for that group. Since Int8 quantization is employed, the number of statistical intervals should exceed 128 to ensure that each group has a corresponding quantization bit during the Saturated Truncation. Saturated Truncation is then utilized as described in Algorithm 1, calculating the truncation thresholds *t* for the activation values of each layer. Following this, the activation values within the interval [−t,t] are quantized using 8-bit Symmetric Uniform Quantization, as specified by Equations (2)–(4). For activation values falling outside the truncation intervals, their quantization bits are assigned to ±127, depending on their sign. The EdgeSAM quantized using the Saturated Truncation strategy is referred to as EntQ-EdgeSAM.
**Algorithm 1:** Saturated Truncation
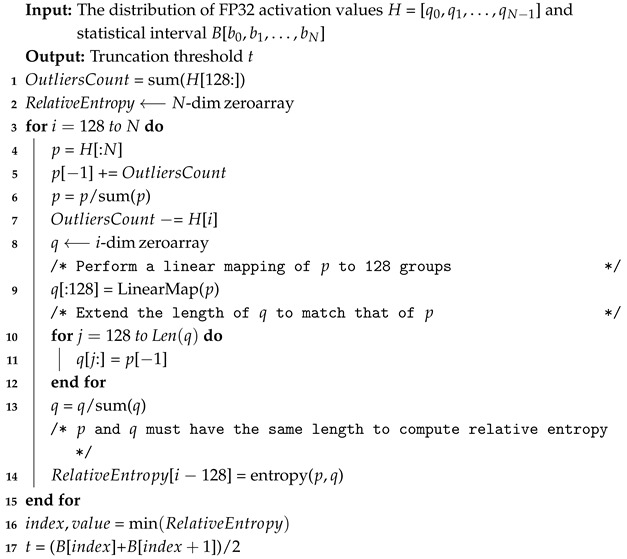



### 2.3. Grasp Pose Determination Module

The target object’s point cloud is required to generate accurate 6D grasp poses. However, directly extracting the target object’s point cloud from the entire environment for specific object grasping is challenging. This requires using specialized point cloud annotation tools for dense and high-quality annotations and training on devices equipped with sufficient computational resources [37]. Furthermore, models involving object point cloud extraction consume significant computational resources, reducing the operational efficiency of the robotic control system.

A PCEM based on DIS principle is proposed to address the aforementioned challenges in this study. The point cloud generation can be simplified to calculate the 3D coordinates of the object point in the camera coordinate system by giving the known pixel coordinates of the object point and the corresponding depth information. The mathematical model for this process is shown in Equation (8):

(8) $$h{\left[\begin{array}{c} u\\ v\\ 1 \end{array}\right]}^{T}=A{\left[\begin{array}{c} x\\ y\\ z\\ 1 \end{array}\right]}_{Cam}$$

where *u*, *v* represent the image pixel coordinates, 1 is the homogeneous coordinate that satisfies the equation, $x_{Cam}$, $y_{Cam}$ and $z_{Cam}$ represent the coordinates of the object point in the camera coordinate system, $A\in R^{3\times 4}$ denotes the camera intrinsic parameter matrix which is fixed for each camera, *h* represents the depth information coordinate, and T represents the transpose of the matrix. Since the depth camera coordinate system is typically aligned with the RGB camera coordinate system in most visual devices, we have $h=z_{Cam}$. Assuming the pixel coordinates of the camera’s optical center are $\left(c_{x},c_{y}\right)$, and the ratio of the focal length to the physical length per unit pixel is $f_{x}$, $f_{y}$, the conversion from the pixel coordinate system to the camera coordinate system can be described by the following equation based on the camera imaging principles:

(9) $$x_{Cam}=\frac{\left(u-c_{x}\right)}{f_{x}}\times h$$

(10) $$y_{Cam}=\frac{\left(u-c_{y}\right)}{f_{y}}\times h$$

The complete mathematical model derived from Equation (11) is as follows:

(11) $$h{\left[\begin{array}{c} u\\ v\\ 1 \end{array}\right]}^{T}=\left[\begin{array}{cccc} f_{x} & 0 & c_{x} & 0\\ 0 & f_{y} & c_{y} & 0\\ 0 & 0 & 1 & 0 \end{array}\right]{\left[\begin{array}{c} x\\ y\\ z\\ 1 \end{array}\right]}_{Cam}$$

Using the per-pixel depth values provided by the depth map and the grid-like pixel coordinates provided by the RGB image, the corresponding 3D coordinates in the camera coordinate system for all pixel nodes are generated utilizing Equation (11). Then, by utilizing the binary mask of the target object, a binary weight *g* is assigned to all pixel nodes of the depth map, transforming Equation (11) into the following form:

(12) $$gh{\left[\begin{array}{c} u\\ v\\ 1 \end{array}\right]}^{T}=\left[\begin{array}{cccc} f_{x} & 0 & c_{x} & 0\\ 0 & f_{y} & c_{y} & 0\\ 0 & 0 & 1 & 0 \end{array}\right]{\left[\begin{array}{c} x\\ y\\ z\\ 1 \end{array}\right]}_{Cam},\;g\in \left\{0,1\right\}$$

When a pixel node belongs to the target object, g=1, and the corresponding spatial point for this pixel node is calculated using Equation (11); conversely, when the pixel node does not belong to the target object, g=0, and the spatial point is solved as $x_{Cam}=y_{Cam}=z_{Cam}=0$, which means the point is located at the camera coordinate origin and considered “suppressed” in the point cloud field. To enhance computational efficiency, Equation (12) is rewritten in matrix multiplication form to simultaneously calculate for all pixel nodes:

(13) $$G⊙H{\left[\begin{array}{c} U\\ V\\ 1 \end{array}\right]}^{T}=\left[\begin{array}{cccc} f_{x} & 0 & c_{x} & 0\\ 0 & f_{y} & c_{y} & 0\\ 0 & 0 & 1 & 0 \end{array}\right]{\left[\begin{array}{c} X\\ Y\\ Z\\ 1 \end{array}\right]}_{Cam},\;G\in \left\{0,1\right\}$$

where $G\in {\left\{0,1\right\}}^{h\times w}$ represents a binary weight matrix, $H\in R^{h\times w}$ represents a depth information matrix, $U,V\in Z^{h\times w}$ represent pixel node matrices, and $X,Y,Z\in R^{h\times w}$ represent spatial information matrices in the camera coordinate system. The operator ⊙ represents element-wise multiplication between matrices.

It should be noted that the proposed Depth Information Suppression (DIS) method does not explicitly apply denoising or filtering operations to the depth map. Instead, the noise robustness is achieved implicitly through the binary suppression mechanism. Since only the pixels belonging to the target object (g=1) contribute to the point cloud reconstruction, most background and boundary noise points are naturally discarded. Moreover, depth noise typically appears in reflective, transparent, or occluded regions that are excluded by the object mask. Therefore, the reconstructed target point cloud is less affected by depth disturbances, providing a cleaner and more consistent geometric representation for 6D grasp pose estimation. This design simplifies the computation while maintaining sufficient accuracy for real-time robotic grasping.

As shown in Figure 1, the point cloud of target object can be calculated using Equation (12) by combining the object mask with the pixel nodes and depth information from the current view’s RGB and depth images. To ensure the generalization capability of the grasping framework, the TPGF need to employ a GPDM with realistic perception capabilities. The AnyGrasp is pre-trained on a large-scale dataset consisting of 268 real scenes with 144 objects. It achieves a 93.3% success rate in household object grasping experiments [3], which is comparable to human experimental group results, making it suitable for integrating into our framework. Additionally, AnyGrasp takes scene point clouds as input but outputs grasp poses for all grabbable objects within the scene rather than for a specific object. Our method addresses this limitation by suppressing points in the scene point cloud that do not belong to the target object, enabling AnyGrasp to generate robust grasp poses specifically for the target object.

## 3. Comparison Experiments and Discussion

### 3.1. Dataset

**Figure 3:**
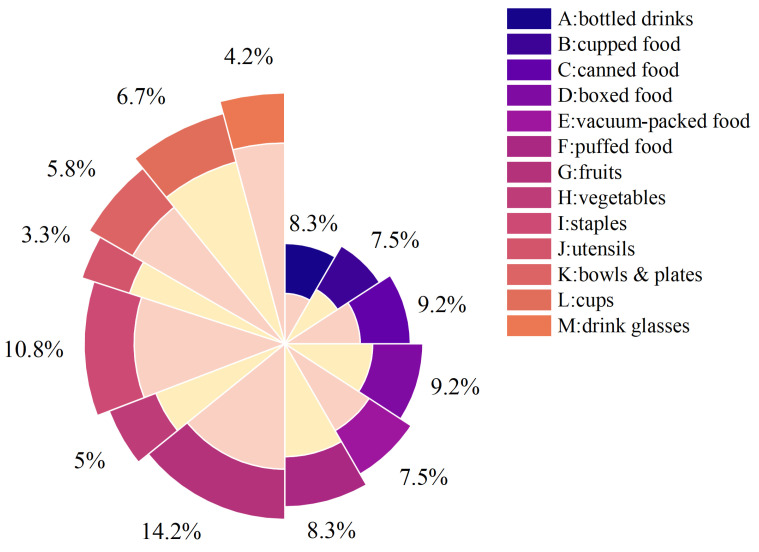
Object category statistics in MessyTable.

(1) **Dataset**: We utilize the real-world dataset MessyTable [38] to conduct experiments to evaluate the TPGF’s generalization and quantization performance. The MessyTable dataset contains 50,211 images with a resolution of 1920×1080, covering 5579 everyday life scenes. Each scene includes nine different viewpoints with dense annotations of 120 household items across 13 major categories. As the dataset did not provide detailed statistics on the number of categories, we first conducted a manual count and plotted Figure 3.

(2) **Dataset Preprocessing**: First, the real detection labels of instances in each image are extracted and categorized. These labels are then fed into SAM-H as box prompts to generate instance masks, which serve as the baseline for mask quality evaluation. For the generalization performance experiments, we randomly divide the dataset into training, validation, and test set. Specifically, the training set accounts for 67% of the total images, the validation set accounts for 17%, and the remaining images are used for model test. To simulate the zero-shot task, two items are randomly selected from each major category as zero-shot objects and excluded from the training and validation set. In the target detection phase of the generalization performance experiment, the task-specific models are trained directly using the detection labels from the training and validation sets. For mask quality evaluation, the masks of instances in the training and validation set are converted to mask label files in COCO format, forming new training and validation set for the segmentation-specific models. Quantization performance experiments are conducted on the entire dataset.

### 3.2. Metrics

In the generalization performance experiment, the Intersection over Union (IoU) of the predicted bounding boxes with the ground truth labels is used as the evaluation metric for detection accuracy. The calculation formula is as follows:

(14) $$IoU_{\mathrm{BBox}}=\frac{B_{Pred}\cap B_{GT}}{B_{Pred}\cup B_{GT}}$$

where $B_{Pred}$ represents the geometric area of the predicted bounding box, and $B_{GT}$ represents the geometric area of the corresponding ground truth bounding box. To analyze the quality distribution of predicted bounding boxes, detection accuracy is evaluated for all instances using mean Average Precision (mAP) at IoU thresholds of 0.5 and 0.95, calculated with the following formula:

(15) $$mAPx=\frac{1}{N}\sum_{i=1}^{N}APx\times 100\%$$

where *x* is 50 or 95, indicating that this detection is considered accurate when the calculated IoU is greater than 0.5 or 0.95, and *N* indicates the total number of categories.

We use the precision/recall/F-measure (P/R/F) metrics to evaluate the matching degree between the predicted mask and baseline when all methods are detected accurately. The Precision, Recall, and F-measure scores are calculated using the following Equation (16) [39]:

(16) $$P=\frac{\sum_{i}|s_{i}\cap g\left(s_{i}\right)|}{\sum_{i}\left|s_{i}\right|},R=\frac{\sum_{i}\left|s_{i}\cap g\left(s_{i}\right)\right|}{\sum_{j}\left|g_{j}\right|},F=\frac{2PR}{P+R}$$

where $s_{i}$ represents the pixel set of the predicted object’s mask, $g(s_{i})$ represents the pixel set of the ground truth mask corresponding to the predicted object, and $g_{j}$ represents the pixel set of the ground truth mask for the object *j*.

In the quantization performance experiment, the IoU of predicted masks with baseline is used as the evaluation metric for quantization performance. The calculation formulas of IoU and mIoU are as follows:

(17) $$IoU_{\mathrm{mask}}=\frac{M_{mask}\cap M_{base}}{M_{mask}\cup M_{base}}$$

(18) $$mIoU=\frac{1}{N}\sum_{i=1}^{N}IoU_{mask_{i}}$$

where $M_{mask}$ represents the pixel area of the predicted mask, and $M_{base}$ represents the pixel area of the corresponding baseline. *i* denotes one of the object categories from ‘A’ to ‘M’.

The Compression Rate (CR) of the TensorRT engine file is also used as evaluation metrics. The formula for calculating the Compression Rate is as follows:

(19) $$Compression\;rate=\frac{Sto_{int8}}{Sto_{fp32}}$$

where $Sto_{int8}$ and $Sto_{fp32}$ represent the storage space usage of the image encoder’s TensorRT engine file before and after quantization, respectively.

### 3.3. Generalization Performance Experiment

To demonstrate the advantages of the proposed TPGF in object perception generalization, experiments compare it against Ceschini et al.’s Mask R-CNN for object perception as a baseline, alongside recent state-of-the-art(SOTA) task-specific models YOLOv8-seg [40], YOLOv9-seg [41], YOLOv10 [42], and YOLOv11-seg [43]. The above preprocessed dataset is used to train on the aforementioned models. The training is performed using an NVIDIA GeForce RTX 2080 Super GPU. The models are trained for 50 epochs with a batch size of 16. Data mosaic is disabled after 35 epochs, the learning rate is set to 0.01, the momentum is 0.937, the consumed memory is 7 GB, the iterative strategy is performed through the SGD optimizer, and each training takes an average time of 12 h. Grounding DINO is neither retrained or fine-tuned on the dataset.

To compare the performance of the task-specific model and our method in zero-shot tasks, experiments on object detection for both regular and zero-shot tasks are conducted using the test set. Regular tasks involve detecting instances present in both the test set and the training or validation sets, while zero-shot tasks detect instances exclusive to the test set. Table 1 shows the detection accuracy of Grounding DINO compared to task-specific models. In the regular tasks, the supervised trained task-specific models have a certain advantage over Grounding DINO under the mAP50 metric, and YOLOv10 has the best performance at 89.75%. Under mAP95, the accuracy of task-specific models drops significantly, with YOLOv11 showing the largest decrease of 61.66% and YOLOv10 the smallest at 36.46%. The proportion of high-quality bounding boxes is 23.51% and 59.37%, respectively. Mask R-CNN demonstrates the same trend as the YOLO series models, showing a significant decline in detection performance for zero-shot tasks. This indicates that the grasping framework proposed by Ceschini et al. [18] is not suitable for zero-shot object perception tasks in everyday environments. Grounding DINO ranks second with the smallest performance drop of 24.16%. It achieves the highest proportion of high-quality bounding boxes, ranking first at 59.77%. Even if Grounding DINO does not carry out additional training or fine-tuning on the MessyTable dataset, its overall detection accuracy is only slightly lower than that of task-specific models trained explicitly on MessyTable. Nevertheless, it demonstrates a greater likelihood of producing high-quality bounding boxes for successfully detected objects, thereby enabling the generation of high-quality masks in subsequent processing steps. In zero-shot tasks, Grounding DINO maintains detection performance comparable to that in regular tasks, while task-specific models all exhibit a drop of over 58%. The highest proportion of high-quality bounding boxes is only 18%, with YOLOv9 reaching the lowest at 10.73%. This demonstrates Grounding DINO’s superior performance in zero-shot tasks compared to task-specific models, achieving stable zero-shot object detection without additional training or fine-tuning.

The experiments further compare the mask P/R/F scores of YOLOv8-seg, YOLOv9-seg, YOLO11-seg, Mask R-CNN and “Grounding DINO + EntQ-EdgeSAM” for accurately detecting the same object at IoU thresholds of 0.5 and 0.95. The results are shown in Table 2.

Except for Mask R-CNN under mAP50, we see that both the task-specific models and “Grounding DINO + EntQ-EdgeSAM” show lower precision than recall, which indicates that both of them suffer from over-segmentation of the scene, but the task-specific models are more serious than our method. Moreover, in zero-shot tasks, task-specific models show further precision degradation. Their F-measure scores significantly reduce compared to “Grounding DINO + EntQ-EdgeSAM”. It should be noted that in the mAP50 experimental group for zero-shot tasks, our method’s results are significantly lower than those in the mAP95 experimental group. This is due to partially overlapping instances. Nevertheless, our method still outperforms task-specific models in terms of P/R/F scores.

Synthesizing the above analysis, it is concluded that task-specific models produce more false positives (where the model predicts a pixel belongs to an object, but it actually belongs to the background in the baseline) compared to “Grounding DINO + EntQ-EdgeSAM”. The false positive cases will introduce ambient noise in the Object Point Cloud Extraction stage, leading to the misjudgment of the grasping pose determination and increasing collision risks during the grasping process. In contrast, “Grounding DINO + EntQ-EdgeSAM” generates fewer false positives and higher-quality masks, resulting in cleaner object point clouds and more accurate grasp poses in the following stage.

Although the MessyTable dataset provides a challenging benchmark encompassing 13 major categories and a total of 120 household items, it fails to fully capture the long-tail distribution of household objects, such as deformable or highly irregular items. It is noteworthy that the TPGF isn’t tailored specifically for MessyTable. Instead, it leverages the open-set object detector Grounding DINO for object perception and the category-agnostic segmentation model EntQ-EdgeSAM to extract pixel-level object features. This design enables the framework to transcend the realm of rigid household items, laying the groundwork for handling irregular or deformable objects.

### 3.4. Quantization Performance Experiment

To demonstrate that EntQ-EdgeSAM exhibits superior quantization performance compared to SAM-H used by Ceschini et al. [18] and Li et al. [20], quantization performance experiments are conducted across the entire MessyTable dataset. Additionally, to explain from the perspective of model architecture why the proposed framework chooses the Saturated Truncation Strategy for quantizing EdgeSAM rather than other SAM variants, we select representative variants from different image encoder structures of SAM for comparison. Specifically, MobileSAM is chosen as the lightweight ViT-based variant, while EfficientViT-SAM-XL0 represents the hybrid architecture with a modified Attention mechanism. EntQ-EdgeSAM belongs to the pure CNN-based variant of SAM. To highlight the advantages of EntQ-EdgeSAM on devices with limited computational power, the experiment employed an NVIDIA GeForce RTX 2080 Super with 8 GB of VRAM as the inference GPU. However, due to insufficient VRAM, SAM-H could not run on such GPUs. Therefore, SAM-B that requires lower computational resource in SAM series models is selected as the baseline. The TensorRT engine file size for SAM-B is 367.8 MB, achieving an average inference time of 61ms on a single 1920×1080 resolution image. In contrast, SAM-H achieves an average inference time of 650ms on an NVIDIA GeForce RTX 4070 Super with 16 GB of VRAM.

**Figure 4:**
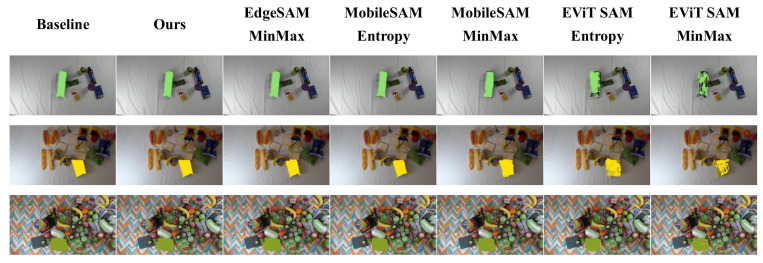
The mask inference results of different SAM variants after quantization. The green and yellow patterns in the image represent object masks.

First, we use the entire MessyTable dataset as the calibration set. Int8 quantization is then performed on the image encoders of the aforementioned models using the Saturated Truncation strategy (Entropy) and Symmetric Uniform Quantization (MinMax) on TensorRT, respectively. The model’s Compression Rate (CR) relative to themselves and to SAM-B (SAM-B-based CR) are calculated after quantization. The prompt encoder and mask decoder utilize the corresponding components of these models. The quantized models are then employed for inference on the entire dataset. The inference is performed on an NVIDIA GeForce RTX 2080 GPU and the average inference time per image of the image encoder is recorded when performing. The corresponding detection ground truth labels for each image serve as box prompts, which are input into the prompt encoder to generate masks. The experiment evaluates the mIoU for all 13 major categories of household items, as shown in Table 3. Figure 4 displays the quantized inference results for different methods.

From a deployment perspective, EntQ-EdgeSAM achieved the fastest average inference time of 3.086 ms (representing a 95% improvement over SAM-B) with a model compression rate of just 3.5% based on SAM-B, fulfilling the real-time and low-memory requirements for robotic grasping tasks. This demonstrates that the EntQ-EdgeSAM resolves the real-time limitations in existing multi-step grasping frameworks—an issue that Ceschini et al. [18] and Li et al. [20] failed to address when embedding SAM-H into their respective frameworks.

Compared to other SAM variants’ combinations with two quantization methods, EntQ-EdgeSAM achieves the best performance across all 13 categories in the dataset, demonstrating the effectiveness of utilizing the Saturated Truncation strategy to pure CNN architectures. Additionally, the combination of EdgeSAM and the MinMax method achieves the second-best results, with its average performance exceeding that of MobileSAM combined with the Saturated Truncation strategy. This improvement can be attributed to the dynamic prompt-in-the-loop strategy proposed by Zhou et al. [28], which allows EdgeSAM to effectively learn the majority of knowledge from SAM. Moreover, the pure CNN architecture of the EdgeSAM image encoder facilitates more uniform data distribution compared to traditional ViT structures, thereby ensuring that quantization using the MinMax method does not result in significant accuracy degradation. MobileSAM retains the ViT architecture, and the presence of the Attention mechanism causes significant data distribution imbalances after the softmax operation, resulting in a severe accuracy drop when the MinMax method is utilized. This observation is consistent with the findings of previous studies [31,32,33]. However, the Saturated Truncation strategy maximizes the alignment between the original and quantized data distributions, significantly improving quantization accuracy compared to the MinMax method. As a result, after utilizing the Saturated Truncation strategy to the MobileSAM image encoder, the mask quality remains at a “usable” level and is comparable to EdgeSAM quantized with the MinMax method in groups A, D, E, G, H, I, K, and L. In Group F, MobileSAM combined with Entropy outperforms EdgeSAM with MinMax. Nevertheless, because EdgeSAM employs the advanced dynamic prompt-in-the-loop strategy, there remains a significant performance gap between MobileSAM and EntQ-EdgeSAM, even when the same quantization method is utilized.

**Figure 5:**
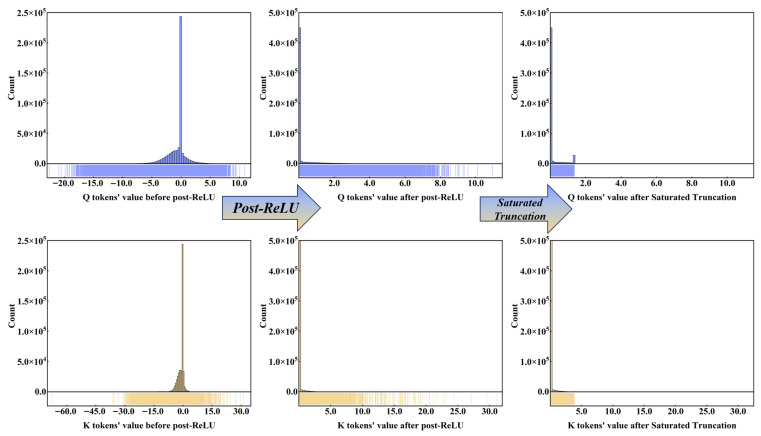
Data distribution of Q and K tokens in the Multi-Scale ReLU Linear Attention of EfficientViT-SAM-XL0 before and after post-RELU calculation. The axis ticks represent the distribution of values. Negative data with valid information are widely distributed in the negative value range, but after post-ReLU calculation, these data are set to zero.

Note that both the Saturated Truncation strategy and MinMax method performed poorly on EfficientViT-SAM-XL0, as shown in Figure 5. We analyze the reasons for the significant accuracy drops in EfficientViT-SAM-XL0 from the Multi-Scale ReLU Linear Attention in its EfficientViT module. During calculations on Q and K tokens, most values cluster around zero after the ReLU calculation. Consequently, when the Saturated Truncation strategy calculates truncation thresholds, some nonzero data containing valid information is excluded from the truncation interval, as it is perceived to increase relative Entropy. After quantization, the distribution of these values deviates significantly from the original distribution, suppressing the Multi-Scale Feature Extraction capability of EfficientViT and leading to a notable drop in inference accuracy.

The accuracy drop with the MinMax method primarily results from all negative values being set to zero after the ReLU calculation, which increases the frequency of data distribution near zero. This leads to a significant distribution between the minimum and maximum values compared to the softmax calculation [31,32,33], resulting in the Multi-Scale ReLU Linear Attention with larger quantization errors than softmax calculation.

## 4. Grasping Experiment and Discussion

### 4.1. Experimental Platform

**Figure 6:**
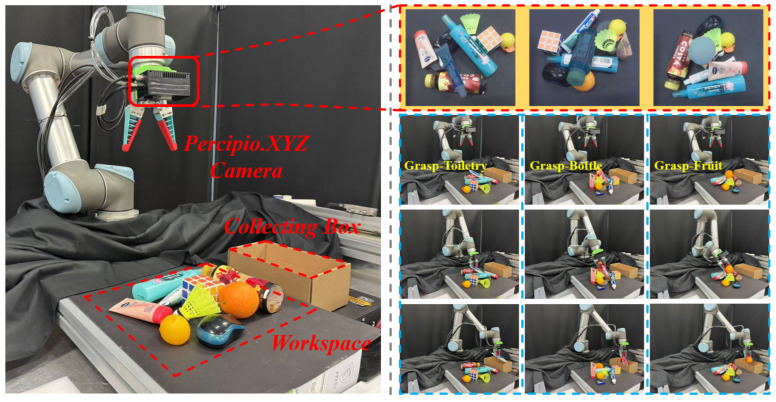
Schematic diagram of the grasping experiment in replicated everyday environments. The left image illustrates the layout of the grasping platform. The upper-right image shows the object perception results of the camera. The lower-right images, from left to right, sequentially depict the grasping experiment processes for toiletries, bottles, and fruits.

To evaluate the effectiveness of the TPGF for zero-shot object grasping in everyday environments, a robotic grasping platform is constructed, as shown on the left side of Figure 6. The platform consists primarily of a UR5 robot and a Percipio.XYZ FS820-E1 3D camera. The camera is mounted on the robot to enable grasping from different viewpoints. The TPGF is deployed on a small industrial control computer with moderate computational resources. The platform captures scene information in real-time by reading RGB and depth images from the camera. Object textual information is obtained by user input. The above information is then fed into the TPGF to output a 6D grasp pose. Finally, control commands are sent from the robot control cabinet to the robot, guiding the robot to the calculated grasp pose and execute the grasping action.

### 4.2. Grasping Experiments in Simulated Everyday Environments and Discussion

**Figure 7:**
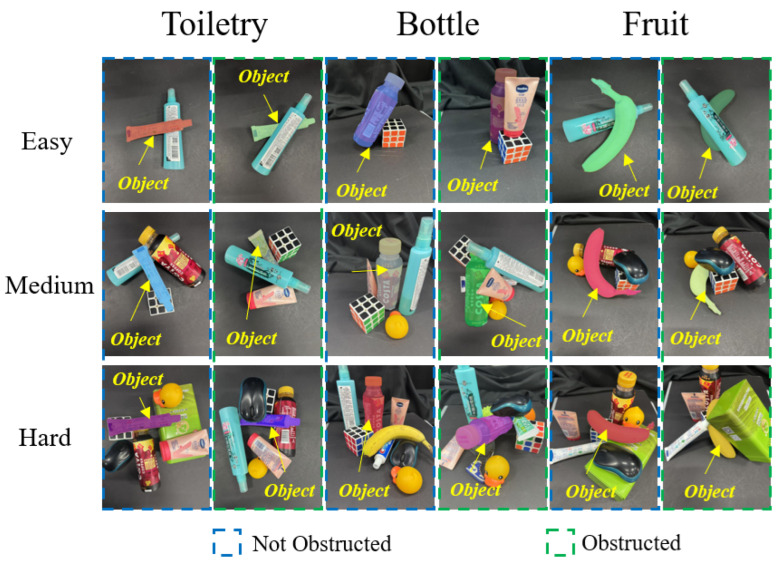
Schematic diagram of object placement in replicated everyday environments.

To realistically simulate everyday environments in the experiment, commonly encountered household items are selected as experimental objects. Among them, three objects with the highest human grasping frequency—fruits, bottles, and toiletries—are chosen as target objects, while the remaining items served as distractions for the object perception and grasp pose determination tasks. Since the camera is mounted on the robot, it is unable to capture the entire scene. Therefore, this experiment only considers the placement of objects within the camera’s field of view. Based on the number of objects and their density, three complexity levels are set: easy, medium, and difficult. For each scene, scenarios with and without target object occlusion are considered, as shown in Figure 7. Additionally, to demonstrate the zero-shot task capability of the grasping framework, the TPGF is neither pre-trained or fine-tuned on any of these objects before the experiment. All objects are randomly selected from their respective categories, and the scene layout is determined solely by the complexity level and the presence or absence of occlusion.

The objective of the grasping experiment is to retrieve target objects from a complex environment and place them into a collecting box. The experimental process is shown on the right side of Figure 6. Considering the impact of point cloud noise caused by the depth map within the mask region on the grasping pose, the TPGF outputs the top 5 grasp poses ranked by grasping scores in each experiment. The robot then sequentially moves to these poses and executes the grasping actions. A case is considered successful if at least one of these grasping poses results in a successful grasp. The experiments are conducted under three levels of scene complexity, with 60, 90, and 90 grasp attempts, respectively. The grasp success rate and the unrecognized rate of target objects are calculated separately for scenarios with and without occlusion, as shown in Table 4.

The experimental results indicate that the proposed TPGF achieves an average of 82.8%, 76.65%, and 76.7% grasping successful rate in three levels of scene complexity. In scenarios where the target object is not occluded, the framework demonstrates a 97.8% object recognition rate. However, in scene with occlusion, the grasping success rate significantly decreases with increasing scene complexity. The grasp success rate experiences a maximum reduction of 20% in difficult scene. The main causes of grasp failure are failed to recognize the target object and the presence of dangerous grasp poses. These issues are most prevalent when the target object is occluded. Excessive occlusion of the target object causes the loss of important features, leading to the failure of the OPM to accurately identify the object. Moreover, the clustering of surrounding obstacles causes the gripper to collide with them during the grasping process, displacing the target object from its initial position. The over-concentration of obstacles also reduces the available grasping space for the target object, forcing the robotic arm to approach the grasp pose in a dangerous configuration, resulting in collisions with the surrounding environment.

Note that some pipeline-based grasping frameworks related to our work [6,18,20,44] also achieves high grasping accuracy using data-driven approaches. However, our TPGF is composed entirely of modules that do not require fine-tuning or training. This significantly reduces training resources and time costs, making it highly advantageous for convenient deployment of the grasping framework on household service robots.

**Figure 8:**
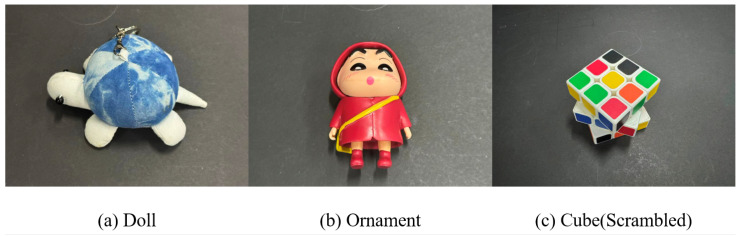
Schematic diagram of certain household items that are difficult to identify or grasp.

The grasping experiments further explored the generalization capabilities of the TPGF when encountering objects that are more difficult to recognize or grasp, as shown in Figure 8.

**Figure 9:**
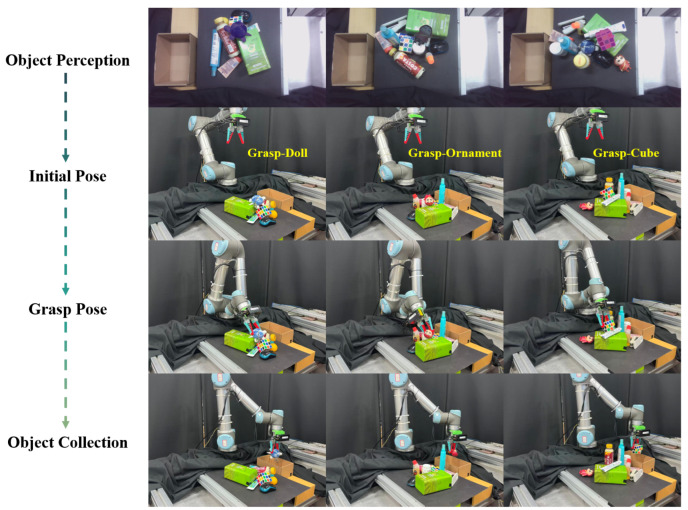
The grasping experiment processes for doll, ornament, and cube.

To highlight the generalization capability of the TPGD grasping framework, the grasping experiments for the aforementioned objects are conducted exclusively in the “hard” scenes. Each of the three objects underwent 30 grasping attempts. The results for object perception and grasping accuracy are shown in Table 5. The grasping experiment workflow is illustrated in Figure 9.

Experimental results demonstrate that the proposed TPGF maintains an object recognition rate exceeding 80% and a grasping success rate of 66.7% for the aforementioned challenging-to-recognize or grasp objects under unobstructed conditions. Even in obstructed scenes, these metrics remain above 66.7% and 53.3%, respectively. Furthermore, none of the aforementioned objects belong to the categories included in MessyTable, which further demonstrates that he TPGF remains effective even when confronted with non-rigid household objects. This proves its robust zero-shot generalization capability and establishes its practical value in addressing the challenges of perception and grasping for household items.

Finally, to highlight the advantages of the proposed TPGF over previous multi-step grasping frameworks, a structured comparison is conducted between our framework and the works by Ceschini et al. [18] and Li et al. [20] As shown in Table 6, the TPGF achieves robust zero-shot generalization and low computational cost by integrating Grounding DINO, EntQ-EdgeSAM, and AnyGrasp into a modular pipeline, thereby addressing the limitations of previous multi-step frameworks.

## 5. Limitations

While the TPGF offers superior zero-shot generalization and deployment efficiency, its performance is constrained by the inherent limitations of its constituent modular components. First, despite OPM’s robust zero-shot generalization capabilities, transparent and mirrored objects will introduce noise when cameras acquire depth information, resulting in imprecise object point clouds. This causes AnyGrasp to generate incorrect 6D grasping poses. For these cases, specialized sensors (e.g., polarization cameras for transparent objects) are required to gather more robust geometric information.

Second, the OPM has performance limits tied to the input quality. Grounding DINO’s performance relies heavily on the quality and specificity of the text prompt. Ambiguous commands (e.g., “pick up the thing”) or commands requiring complex semantic reasoning (e.g., “pick up the item used for writing”) can lead to incorrect bounding box detection. To overcome semantic ambiguity, future work involves integrating VLA backbones for improved scene understanding and disambiguation, allowing the TPGF to ground the command in broader scene context.

## 6. Conclusions

In this study, a TPGF is proposed to successfully address the core challenges facing household service robots: zero-shot grasping in cluttered environments and deployment efficiency under resource constraints. The TPGF is a modular framework characterized by its zero-shot, no-training requirement. It leverages advanced foundational general models for object perception and robust 6D pose estimation. Key findings demonstrate the framework’s effectiveness across two primary domains: generalization and efficiency. The OPM exhibited superior zero-shot generalization, significantly outperforming task-specific baselines in high-quality object detection and mask generation. Crucially, the proposed Saturated Truncation strategy achieved a massive efficiency gain. As a result, the EntQ-EdgeSAM model runs 95% faster than SAM used in existing multi-step grasping frameworks while maintaining high accuracy. The TPGF achieved high grasping success rates in replicated everyday environments. This conclusively validates its robustness and practical value, establishing a high-efficiency baseline for robotic deployment in resource-limited settings. The TPGF provides an efficient, geometrically explicit pipeline.

## Figures and Tables

**Table 1:** Detection accuracy results of other methods and GroundingDINO on the dataset. Bold text indicates the maximum value (optimal value) in the experimental group.

Methods	Regular Tasks		Zero-Shot Tasks	
	mAP50	mAP95	mAP50	mAP95
YOLOv8-seg	80.99%	19.04%	22.18%	2.66%
YOLOv9-seg	87.87%	29.84%	9.17%	0.99%
YOLOv10	**89.75%**	**53.29%**	11.28%	2.03%
YOLOv11-seg	79.45%	17.79%	20.68%	2.55%
Mask R-CNN	85.01%	21.90%	19.46%	2.63%
Grounding DINO	60.05%	35.89%	**58.27%**	**34.20%**

**Table 2:** Comparisons of P/R/F scores between other methods and “Grounding DINO + EntQ-EdgeSAM”. Bold text indicates the maximum value (optimal value) in the experimental group.

Methods	P/R/F Scores	Regular Tasks		Zero-Shot Tasks	
		mAP50	mAP95	mAP50	mAP95
	P	0.847	0.901	0.513	0.916
YOLOv8-seg	R	0.926	0.979	0.638	0.980
	F	0.884	0.938	0.568	0.946
	P	0.864	0.910	0.510	0.915
YOLOv9-seg	R	0.927	0.981	0.578	0.976
	F	0.894	0.944	0.542	0.944
	P	0.843	0.900	0.508	0.915
YOLOv11-seg	R	0.930	0.979	0.590	0.975
	F	0.884	0.937	0.545	0.944
	P	**0.904 **	0.951	0.526	0.940
Mask R-CNN	R	0.889	0.959	0.562	0.971
	F	0.896	0.954	0.543	0.955
	P	0.875	**0.952**	**0.706**	**0.944**
Ours	R	**0.933**	**0.981**	**0.845**	**0.984**
	F	**0.903**	**0.966**	**0.769**	**0.963**

**Table 3:** Quantization performance results of different SAM variants after quantization. The A–M group represents the 13 major categories in the dataset. CR denotes the model compression rate before and after quantization, while SAM-B based CR indicates the compression rate of the quantized model relative to SAM-B. The TensorRT engine file storage space usage of SAM-B is 367.8 MB. The inference time of SAM-B is 61 ms. Bold text indicates the best value in each group, bold and underlined text indicates the better value in the comparison between “EdgeSAM + MinMax” and “MobileSAM + Entropy,” and the tilde underlined indicates equality between them.

Methods	Ours	Ed-SAM MinMax	Mo-SAM Entropy	Mo-SAM MinMax	EViT-SAM Entropy	EViT-SAM MinMax
Groups						
A	**0.893**	0.838	**0.844**	0.692	0.525	0.502
B	**0.893**	**0.851**	0.848	0.741	0.574	0.527
C	**0.888**	**0.848**	0.840	0.716	0.581	0.542
D	**0.878**	**0.823**	**0.823**	0.685	0.536	0.504
E	**0.848**	0.792	**0.793**	0.651	0.488	0.456
F	**0.887**	0.815	**0.853**	0.682	0.483	0.462
G	**0.918**	0.882	**0.883**	0.760	0.596	0.555
H	**0.917**	0.886	**0.887**	0.768	0.573	0.533
I	**0.884**	**0.841**	0.826	0.724	0.567	0.515
J	**0.741**	**0.659**	0.596	0.380	0.279	0.260
K	**0.746**	**0.628**	0.617	0.493	0.384	0.351
L	**0.914**	0.872	**0.873**	0.751	0.592	0.540
M	**0.864**	**0.822**	0.773	0.674	0.605	0.566
CR	0.437	0.389	0.403	0.441	**0.234**	**0.234**
SAM-B-based CR	0.035	**0.031**	0.043	0.047	0.329	0.329
Inference time	**3.086 ms**	3.168 ms	5.757 ms	5.725 ms	3.500 ms	3.432 ms

**Table 4:** Object perception and grasping accuracy results in different replicated everyday environments.

Scene	Not Obstructed			Obstructed		
	Grasped Total	Success Rate	Recognition Rate	Grasped/ Total	Success Rate	Recognition Rate
Easy	40/45	88.9%	97.8%	11/15	73.3%	93.3%
Medium	38/45	84.4%	97.8%	31/45	68.9%	86.7%
Difficult	39/45	86.7%	97.8%	30/45	66.7%	86.7%

**Table 5:** Object perception and grasping accuracy results of doll, ornament and cube.

Scene	Not Obstructed			Obstructed		
	Grasped Total	Success Rate	Recognition Rate	Grasped/ Total	Success Rate	Recognition Rate
Easy	12/15	80%	86.7%	8/15	53.3%	66.7%
Medium	13/15	86.7%	86.7%	11/15	73.3%	80%
Difficult	10/15	66.7%	80%	8/15	53.3%	66.7%

**Table 6:** Structured comparison between prior multi-step grasping frameworks and the proposed TPGF.

Framework	Object Perception	Segmentation	Grasp Generation	Zero-Shot Capability	Computational Cost
Ceschini et al. [18]	Mask R- CNN (close-set detector)	SAM-H	KNN + Theil-Sen algorithm (heuristic grasping method)	Limited (uses a close-set detector)	High (uses SAM- H, 0.5 s on GPU)
Li et al. [20]	Grounding DINO	SAM-H	Sim-Suction-Point net (trained on a synthetic dataset)	Moderate	High (also uses SAM-H)
**TPGF (ours)**	**Grounding** **DINO (open-set** **object detector)**	**EntQ-** **EdgeSAM**	**AnyGrasp (robust** **6D grasping pose** **estimator)**	**Strong**	**Low (uses EntQ-** **EdgeSAM, 3 ms** **on GPU)**

## Data Availability

The data presented in this study are available on request from the corresponding author since some of the experimental data which contains trade secrets of the enterprise is not suitable for public release.
